# Circulating extracellular microvesicles associated with electronic cigarette use increase endothelial cell inflammation and reduce nitric oxide production

**DOI:** 10.1113/EP091715

**Published:** 2024-08-02

**Authors:** Nicholas G. Evanoff, Donald R. Dengel, Kelly A. Stockelman, Hannah Fandl, Noah M. DeSouza, Jared J. Greiner, Sheena R. Dufresne, Michael Kotlyar, Vinicius P. Garcia

**Affiliations:** ^1^ School of Kinesiology University of Minnesota Minneapolis Minnesota USA; ^2^ Center for Pediatric Obesity Medicine University of Minnesota Medical School Minneapolis Minnesota USA; ^3^ Department of Integrative Physiology University of Colorado Boulder Colorado USA; ^4^ Department of Experimental and Clinical Pharmacology University of Minnesota Minneapolis Minnesota USA

**Keywords:** electronic cigarette, endothelial cell, endothelial nitric oxide synthase, inflammation, microvesicle

## Abstract

**Abstract:**

The purpose of this study was to determine the effect of circulating microvesicles isolated from chronic electronic (e‐)cigarette users on cultured human umbilical vein endothelial cell (HUVEC) expression of nuclear factor‐κB (NF‐κB), cellular cytokine release, phosphorylation of endothelial nitric oxide synthase (eNOS) and NO production. The HUVECs were treated with microvesicles isolated via flow cytometry from nine non‐tobacco users (five male and four female; 22 ± 2 years of age) and 10 e‐cigarette users (six male and four female; 22 ± 2 years of age). Microvesicles from e‐cigarette users induced significantly greater release of interleukin‐6 (183.4 ± 23.6 vs. 150.6 ± 15.4 pg/mL; *P* = 0.002) and interleukin‐8 (160.0 ± 31.6 vs. 129.4 ± 11.2 pg/mL; *P* = 0.01), in addition to expression of p‐NF‐κB p65 (Ser536) (18.8 ± 3.4 vs. 15.6 ± 1.5 a.u.; *P* = 0.02) from HUVECs compared with microvesicles from non‐tobacco users. Nuclear factor‐κB p65 was not significantly different between microvesicles from the non‐tobacco users and from the e‐cigarette users (87.6 ± 8.7 vs. 90.4 ± 24.6 a.u.; *P* = 0.701). Neither total eNOS (71.4 ± 21.8 vs. 80.4 ± 24.5 a.u.; *P* = 0.413) nor p‐eNOS (Thr495) (229.2 ± 26.5 vs. 222.1 ± 22.7 a.u.; *P* = 0.542) was significantly different between microvesicle‐treated HUVECs from non‐tobacco users and e‐cigarette users. However, p‐eNOS (Ser1177) (28.9 ± 6.2 vs. 45.8 ± 9.0 a.u.; *P *< 0.001) expression was significantly lower from e‐cigarette users compared with non‐tobacco users. Nitric oxide production was significantly lower (8.2 ± 0.6 vs. 9.7 ± 0.9 μmol/L; *P* = 0.001) in HUVECs treated with microvesicles from e‐cigarette users compared with microvesicles from non‐tobacco users. This study demonstrated increased NF‐κB activation and inflammatory cytokine production, in addition to diminished eNOS activity and NO production resulting from e‐cigarette use.

**Highlights:**

- **What is the central question of this study?**
  Circulating microvesicles contribute to cardiovascular health and disease via their effects on the vascular endothelium. The impact of electronic (e‐)cigarette use on circulating microvesicle phenotype is not well understood.
- **What is the main finding and its importance?**
  Circulating microvesicles from e‐cigarette users increase endothelial cell inflammation and impair endothelial nitric oxide production. Endothelial inflammation and diminished nitric oxide bioavailability are central factors underlying endothelial dysfunction and, in turn, cardiovascular disease risk. Deleterious changes in the functional phenotype of circulating microvesicles might contribute to the reported adverse effects of e‐cigarette use on cardiovascular health.

## 1 INTRODUCTION

Despite regulatory efforts to decrease the use of electronic nicotine delivery systems, electronic (e‐)cigarette use continues to rise, especially amongst adolescents and young adults (McCauley et al., 2022). Although initially proposed to be less harmful than combustible cigarettes, health concerns have emerged regarding e‐cigarette use related to cardiopulmonary disease (Jonas, 2022; McAlinden et al., 2020), chronic kidney disease (Scharf et al., 2022), bone health (Nicholson et al., 2022) and sleep disorders (Brett et al., 2020). Indeed, a recent position statement from the American Heart Association (Singh et al., 2019) has stressed that the potential vascular consequences of acute and chronic e‐cigarette use are not benign. However, the mechanisms underlying e‐cigarette‐associated vascular dysfunction and risk are not well understood. For example, although increased vascular inflammation and diminished endothelial nitric oxide (NO) production have been associated with e‐cigarette use, the causes of this dysfunction are unclear (Damay et al., 2022).

Extracellular microvesicles are small (100−1000 nm in diameter) anucleate membranous vesicles released into the circulation from various cell types, including platelets, red blood cells and endothelial cells, in response to physiological and pathological processes, such as shear stress, differentiation, activation and apoptosis. Regardless of their cellular origin, circulating microvesicles have been shown to initiate the atherosclerotic process by triggering and promoting vascular inflammation, damage and dysfunction (Badimon et al., 2017; Boulanger et al., 2017; Hijmans et al., 2019; Lovren & Verma, 2013). Moreover, extracellular microvesicles have been shown to predict the presence of vascular disease, recurrent hospitalization and all‐cause mortality in a variety of cardiovascular diseases (Bryl‐Górecka et al., 2021; Kanhai et al., 2013; Li et al., 2021; Sun et al., 2022; Wang et al., 2014; Yuan et al., 2022). Although circulating extracellular microvesicles have been shown to be higher in e‐cigarette users (Cardenas et al., 2023; Staudt et al., 2018), it has not been established whether the circulating microvesicle milieu associated with e‐cigarette use is deleterious to vascular endothelial cell function.

Accordingly, the experimental aim of this study was to determine the effect of circulating microvesicles on endothelial cell inflammation and NO production. We tested the hypothesis that circulating microvesicles isolated from chronic e‐cigarette users would promote increased endothelial cell inflammation and impair endothelial NO synthase (eNOS) activation, resulting in diminished NO production. To address our hypothesis, human umbilical vein endothelial cells (HUVECs) were cultured and treated with circulating microvesicles isolated from chronic e‐cigarette users and non‐tobacco users to determine the effects of e‐cigarettes on intracellular expression of the inflammatory mediator nuclear factor‐κB (NF‐κB) and corresponding cellular cytokine release, in addition to phosphorylation of eNOS and corresponding NO production.

## 2 MATERIALS AND METHODS

### 2.1 Ethical approval

This study conformed to the standards set by the *Declaration of Helsinki*, except for registration in a database, and was approved by the University of Minnesota institutional review board (IRB STUDY00004169). Written informed consent was obtained from all participants. The University of Minnesota's Human Research Protection Program (HRPP) provided oversight for this study.

### 2.2 Subjects

Nineteen young adults (18–25 years of age), were studied: nine non‐tobacco users (neither e‐cigarette nor tobacco users; five male and four female; 22 ± 2 years of age; body mass index, 25.0 ± 3.1 kg/m^2^; blood pressure, 129/69 ± 15/9 mmHg) and 10 e‐cigarette users (six male and four female; 22 ± 2 years of age; body mass index, 23.9 ± 3.0 kg/m^2^; blood pressure, 119/67 ± 9/7 mmHg). All participants were not taking medications likely to interfere with the study aims and were free of cardiometabolic abnormalities or overt disease. The e‐cigarette users reported using e‐cigarettes at least five times/day over the past year, with no exposure to other tobacco products. A NicAlert urine cotinine test (NicAlert; Nymox Corporation) was administered to all participants, with a score of at least three required for inclusion in the e‐cigarette group and a score of zero or one required for the non‐smoking group (Gaalema et al., 2011). Non‐use of combustible cigarettes was confirmed with exhaled carbon monoxide concentration of ≤5 p.p.m. (Micro+ Smokerlyzer; Bedfont Scientific Ltd). Within the e‐cigarette use group: three adults used JUUL brand e‐cigarettes (mint flavour); two used Suorin Air brand (fruit flavours); one used Caliburn brand (blue raspberry flavour); one used Smok brand (fruity flavours); one used Novo fruity juice; and two used modified custom boxes (various flavours). All products contained various concentrations of nicotine and various wattage. The average duration of e‐cigarette use was 2.4 years. This study was conducted as part of a larger study evaluating the influence of e‐cigarette use on general vascular health parameters, and the volunteers included took part in a series of studies associated with this trial. However, the experimental aims, a priori hypotheses and associated data presented herein are new and address independent research questions. The study is not registered in a research database.

### 2.3 Microvesicle isolation

Microvesicle isolation was performed as previously described (Brewster et al., 2020). Briefly, venous blood from an antecubital vein was collected in sodium citrate tubes, and differential centrifugation was used to pellet extracellular vesicles. Pelleted extracellular vesicles were then suspended in medium (vascular cell basal medium, no. PCS‐100‐030; fetal bovine serum, no. PCS‐999‐010, ATCC, Manassas, VA, USA; penicillin–streptomycin, no. 15070‐063, Gibco, ThermoFisher, Waltham, MA, USA), sorted and collected by fluorescence‐activated cell sorting (BD FACSAria instrument). The microvesicle size threshold was established using calibrator beads (Megamix‐Plus SSC, Biocytex, Marseille, France), and only events >0.2 and <0.8 μm in size were sorted and collected (Supporting Information, Figure S1). The resulting microvesicles were resuspended in a similar culture medium (vascular cell basal medium, no. PCS‐100‐030; fetal bovine serum, no. PCS‐999‐010, ATCC; penicillin–streptomycin, no. 15070‐063, Gibco) and stored at −80°C (∼6 months) for later use in cell experiments. The microvesicle samples were subjected to a single freeze–thaw cycle 2 h before the microvesicle treatment.

### 2.4 Cell culture and microvesicle treatment

Human umbilical vein endothelial cells (HUVECs; Life Technologies, Thermo Fisher Scientific, Waltham, MA, USA) were cultured in endothelial growth media (ATCC) supplemented with 100 U/mL penicillin and 100 μg/mL streptomycin in standard cell culture conditions (Hijmans et al., 2019). After the third passage, cells were harvested, counted (Z1 Coulter Counter, Beckman Coulter, USA), and 1 × 10^6^ cells were seeded into six‐well tissue culture plates. Thereafter, HUVECs were treated with media containing an equal concentration of microvesicles from either non‐users or e‐cigarette users at a 2:1 (microvesicles:HUVECs) ratio for 24 h. To evaluate whether the cellular effects of microvesicles are mediated through endocytic pathways, microvesicle‐treated cells were also precultured with a cocktail of endocytosis inhibitors composed of: [5‐(*N*‐ethyl‐*N*‐isopropyl) amiloride (0.8 μM; #1154‐25‐2, MilliporeSigma, Burlington, MA, USA), filipin (1.5 μM; #480‐49‐9, MilliporeSigma) and chlorpromazine (7 μM; #69‐09‐0, MilliporeSigma)] for 2 h at 37°C before treatment with microvesicles. In addition, microvesicle‐treated cells were cultured in the absence or presence of the NF‐κB inhibitor IκB kinase (IKK)‐16 (400 nM, IKK inhibitor VII, MilliporeSigma) for 24 h. The concentration of all inhibitors used provided maximal inhibition without cytotoxic or apoptotic effects. The cytotoxicity of each inhibitor was assessed by MTT assay (Ghasemi et al., 2021) (Supporting Information, Figure S2).

### 2.5 Intracellular protein expression

Whole‐cell lysates were obtained from microvesicle‐treated HUVECs for the quantification of intracellular proteins as previously described by researchers in our laboratory (Hijmans et al., 2019). Protein expression was measured by capillary electrophoresis immunoassay (Wes, ProteinSimple, Santa Clara, CA, USA). All protein expression was normalized to total protein in the sample and presented as arbitrary units (a.u.). We used rabbit primary antibodies against NF‐κB p65 (no. D14E12), phosphorylated (p)‐NF‐κB p65 (Ser536) (no. 93H1) (all at a dilution of 1:250) (Cell Signaling Technologies, Danvers, MA, USA), endothelial nitric oxide synthase (eNOS; no. PA1‐037), phosphorylated (p)‐eNOS (Ser1177; no. PA5‐35879) and p‐eNOS (Thr495; no. PA5‐17706) (diluted 1:50, 1:250 and 1:250, respectively; Thermo Fisher Scientific). Initial titrations were performed to optimize the antibody and total protein concentration for each protein target of interest. Representative histograms of the proteins of interest are shown in Figure 1.

FIGURE 1Representative protein histograms of immunodetection by capillary electrophoresis immunoassay for nuclear factor‐κB (NF‐κB; a), phosphorylated nuclear factor‐κB (p‐NF‐κB; b), endothelial nitric oxide synthase (eNOS; c), phosphorylated endothelial nitric oxide synthase [p‐eNOS (Ser1177); d] and p‐eNOS (Thr495) (e).

**Figure:**
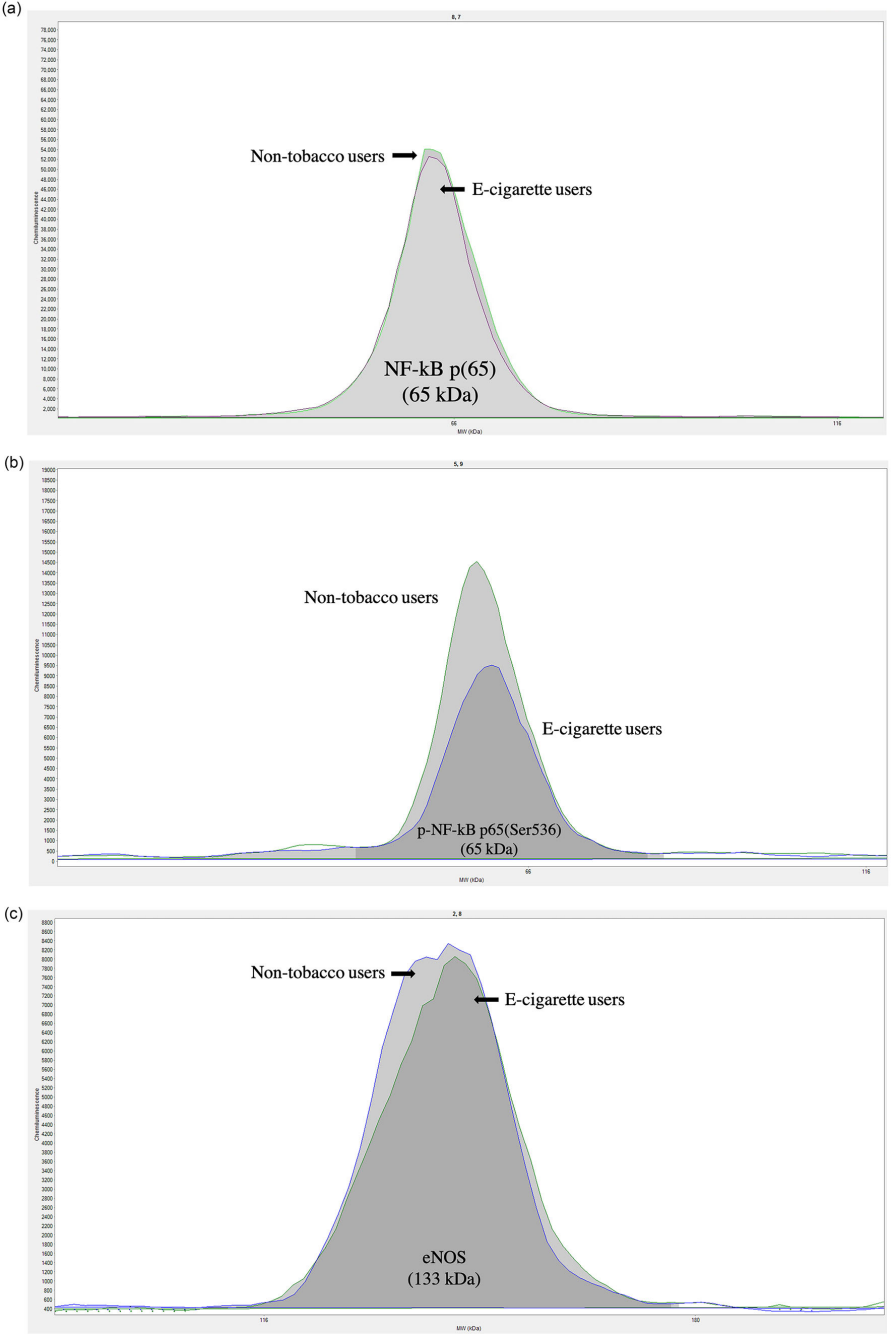

**Figure:**
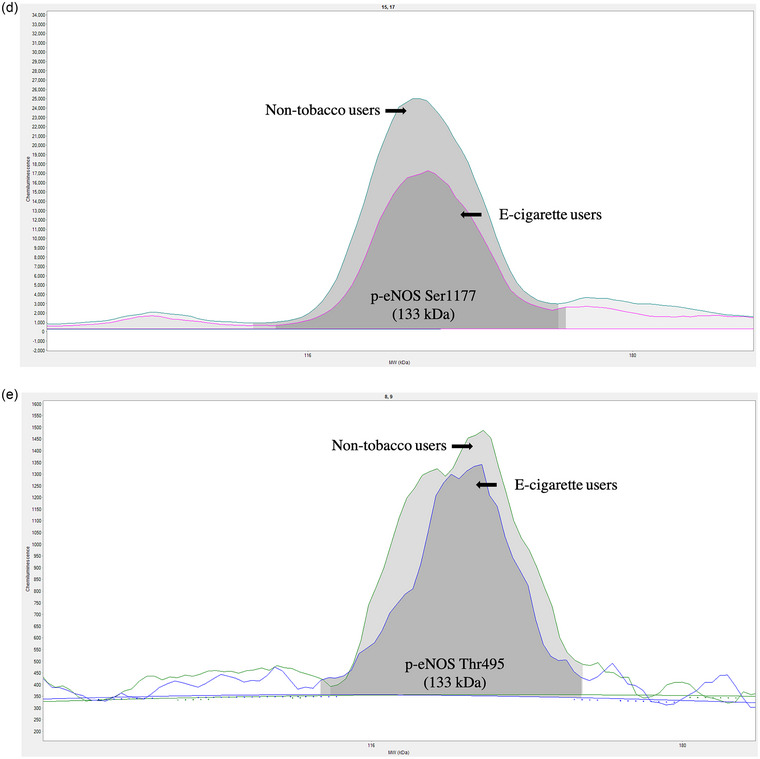

### 2.6 Cytokine release and nitric oxide production

Release of interleukin (IL)‐6 and IL‐8 into the cell supernatant was determined using a chemiluminescent enzyme‐linked immunosorbent assay (R&D Systems, Minneapolis, MN, USA). To assess NO production, a total NO and nitrate/nitrite parameters assay kit (R&D Systems, Minneapolis, MN, USA) was used to quantify total nitrite in the supernatant. Intra‐assay coefficients of variation were <10% for each assay.

### 2.7 Statistical analysis

The distribution of the data was assessed by the Shapiro–Wilk test and the homogeneity of variances by the Levene test. Differences in cellular outcome variables were determined by one‐way ANOVA and were indicated by a significant *F*‐value. *Post hoc* tests with Bonferroni correction for multiple comparisons were performed. Data are presented as means ± SD. Statistical significance was set a priori at *P* < 0.05.

## 3 RESULTS

### 3.1 Endothelial cell inflammation

**FIGURE 2:**
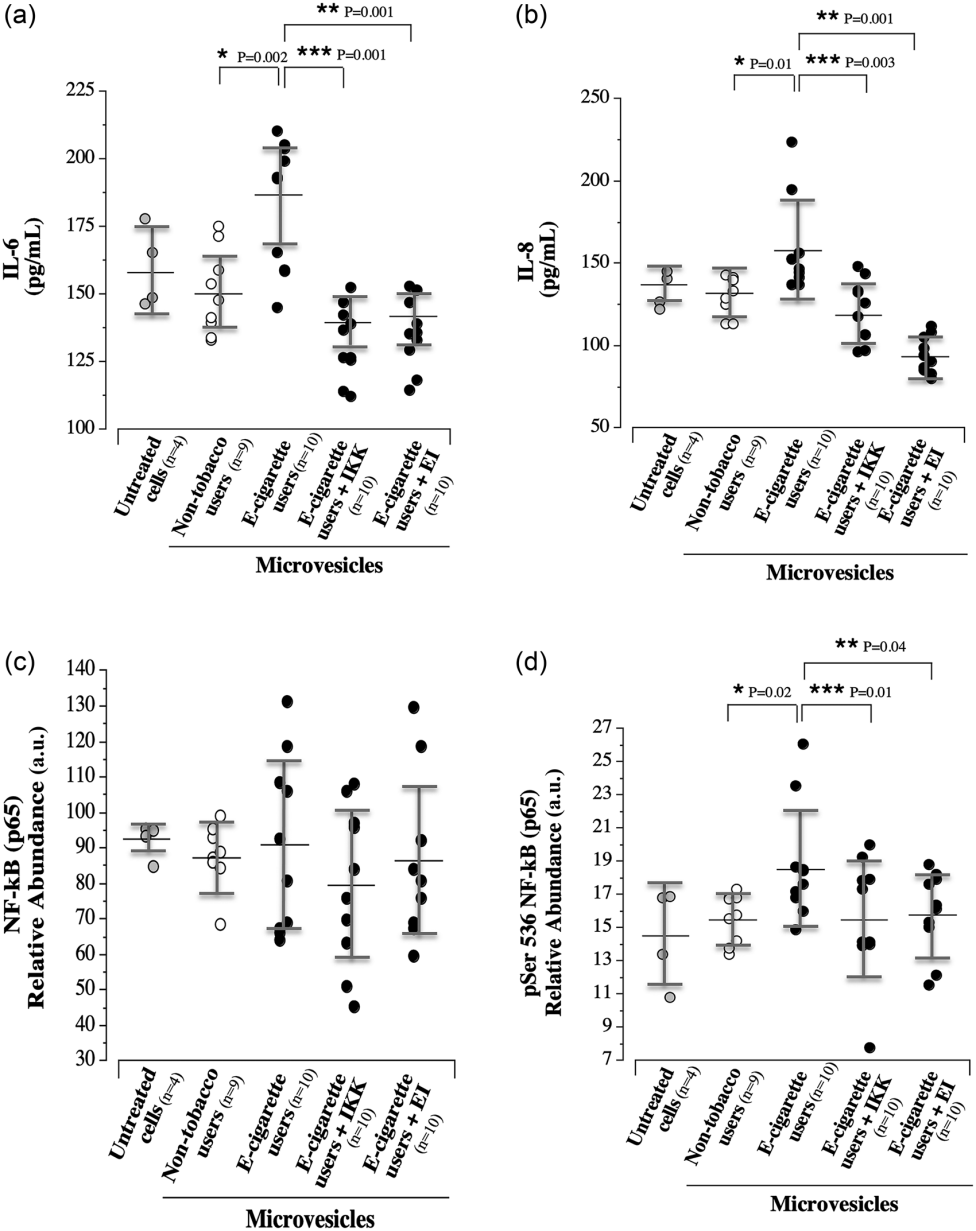
(a) Endothelial cell release of IL‐6 in response to untreated cells (*n* = 4) and treatment with microvesicles from non‐smokers (*n* = 9) and electronic (e‐)cigarette smokers (*n* = 10), in addition to responses in presences of IKK‐16 blockade (*n* = 10) and EI (*n* = 10). (b) Endothelial cell release of IL‐8 in response to untreated cells (*n* = 4) and treatment with microvesicles from non‐smokers (*n* = 9) and e‐cigarette smokers (*n* = 10), in addition to responses in the presence of IKK‐16 blockade (*n* = 10) and EI (*n* = 10). (c) Intracellular expression of NF‐κB p65 in response to untreated cells (*n* = 4) and treatment with microvesicles from non‐smokers (*n* = 9) and e‐cigarette smokers (*n* = 10), in addition to responses in the presence of IKK‐16 blockade (*n* = 10) and EI (*n* = 10). (d) Intracellular expression of p‐NF‐κB p65 (Ser536) in response to untreated cells (*n* = 4) and treatment with microvesicles from non‐smokers (*n* = 9) and e‐cigarette smokers (*n* = 10), in addition to responses in the presence of IKK‐16 blockade (*n* = 10) and EI (*n* = 10). The mean value (black line) and SD (grey brackets) are denoted for each group. Abbreviations: a.u., arbitrary units; EI, endocytosis inhibitors; IKK‐16, IκB kinase; IL, interleukin; NF‐κB, nuclear factor‐κB.

The effect of circulating microvesicles on HUVEC cytokine release and expression of NF‐κB p65 (Ser536) and p‐NF‐κB p65 (Ser536; active NF‐κB) is shown in Figure 2. Microvesicles isolated from e‐cigarette users induced significantly greater release of IL‐6 (183.4 ± 23.6 vs. 150.6 ± 15.4 pg/mL; *F* = 18.9; *P* = 0.002) and IL‐8 (160.0 ± 31.6 vs. 129.4 ± 11.2 pg/mL; *F* = 17.9; *P* = 0.01) from HUVECs compared with microvesicles from non‐tobacco users. Intracellular expression of NF‐κB p65 was not significantly different between cells treated with microvesicles from the non‐tobacco users and microvesicles from the e‐cigarette users (87.6 ± 8.7 vs. 90.4 ± 24.6 a.u.; *P* = 0.70). However, expression of p‐NF‐κB p65 (Ser536) was significantly higher (*F* = 2.7; *P* = 0.02) in HUVECs treated with microvesicles from e‐cigarette users (18.8 ± 3.4 a.u.) compared with microvesicles from non‐tobacco users (15.6 ± 1.5 a.u.). Inhibiting NF‐κB activation with IKK‐16 significantly reduced IL‐6 (183.4 ± 23.6 vs. 132.3 ± 13.5 pg/mL; *F* = 18.9; *P *< 0.001) and IL‐8 (160.0 ± 31.6 vs. 120.8 ± 18.7 pg/mL; *F* = 17.9; *P* = 0.003) in cells treated with microvesicles from the e‐cigarette users. Although IKK‐16 had no significant effect on total NF‐κB expression (90.4 ± 24.6 vs. 79.6 ± 22.3 a.u.; *P* = 0.13), p‐NF‐κB (Ser536) expression was ∼40% lower (15.6 ± 3.6 vs. 18.8 ± 3.4 a.u.; *F* = 2.7; *P* = 0.01) in the cells co‐treated with microvesicles from the e‐cigarette users and IKK‐16 (Figure 2). In addition, inhibition of endocytosis blunted the effect of microvesicles from e‐cigarette users on IL‐6 (135.4 ± 12.8 vs. 183.4 ± 23.6 pg/mL; *F* = 18.9; *P *< 0.001), IL‐8 (94.2 ± 15.2 vs. 160.0 ± 31.6 pg/mL; *F* = 17.9; *P *< 0.001) and p‐NF‐κB (Ser536) expression (15.8 ± 2.6 vs. 18.8 ± 3.4 a.u.; *F* = 2.7; *P* = 0.04) but had no significant effect on total NF‐κB expression (90.4 ± 24.6 vs. 87.5 ± 21.2 a.u.; *P* = 0.21) (Figure 2). There were no significant differences in cytokine release and expression of NF‐κB p65 and phosphorylated NF‐κB p65 between the untreated control cells and cells treated with microvesicles from non‐tobacco users [IL‐6: 159.7 ± 15.0 vs. 150.6 ± 15.4 pg/mL, *P* = 0.34 (Figure 2a); IL‐8: 136.6 ± 10.9 vs. 129.4 ± 11.2 pg/mL, *P* = 0.542 (Figure 2b); NF‐κB p65: 92.2 ± 4.9 vs. 87.6 ± 8.7 a.u., *P* = 0.36 (Figure 2c); and p‐NF‐κB p65: 14.5 ± 2.9 vs. 15.6 ± 1.5 a.u., *P* = 0.37 (Figure 2d)].

### 3.2 Endothelial nitric oxide synthase expression and NO production

**FIGURE 3:**
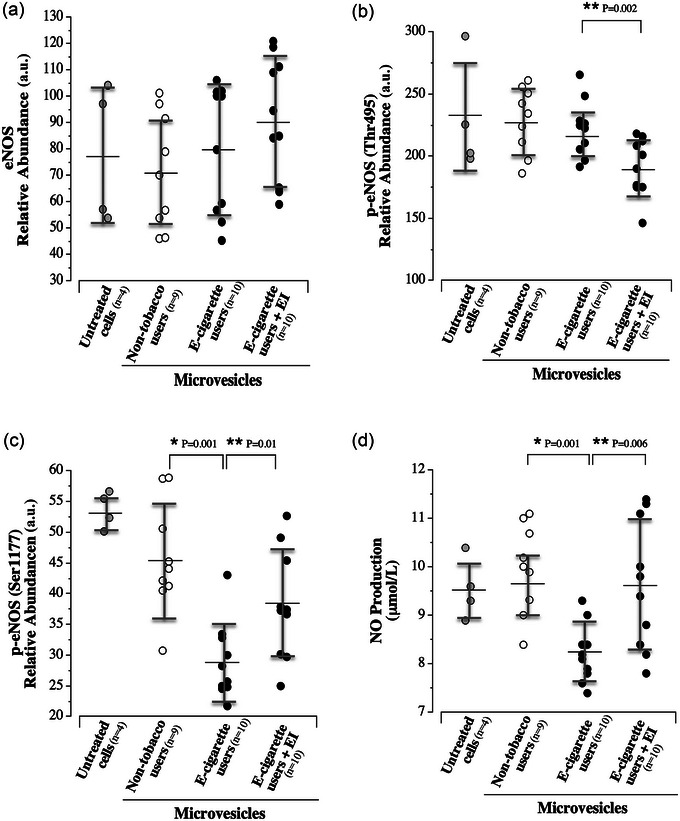
(a) Endothelial cell expression of total eNOS in response to untreated cells (*n* = 4) and treatment with microvesicles from non‐smokers (*n* = 9) and electronic (e‐)cigarette smokers (*n* = 10) and EI (*n* = 10). (b) Endothelial cell expression of total p‐eNOS (Ser1177) in response to untreated cells (*n* = 4) and treatment with microvesicles from non‐smokers (*n* = 9) and e‐cigarette smokers (*n* = 10) and EI (*n* = 10). (c) Endothelial cell expression of total p‐eNOS (Thr495) in response to untreated cells (*n* = 4) and treatment with microvesicles from non‐smokers (*n* = 9) and e‐cigarette smokers (*n* = 10) and EI (*n* = 10). (d) Endothelial cell expression of total NO production in response to untreated cells (*n* = 4) and treatment with microvesicles from non‐smokers (*n* = 9) and e‐cigarette smokers (*n *= 10) and EI (*n* = 10). The mean value (black line) and SD (grey brackets) are denoted for each group. Abbreviation: AU, arbitrary units; EI, endocytosis inhibitors; eNOS, endothelial nitric oxide synthase.

Cellular expression of eNOS, p‐eNOS (Ser1177) and p‐eNOS (Thr495) and NO production are shown in Figure 3. Total eNOS expression (71.4 ± 21.8 vs. 80.4 ± 24.5 a.u.; *P* = 0.413; Figure 3a) and p‐eNOS (Thr495) expression (229.2 ± 26.5 vs. 222.1 ± 22.7 a.u.; *P* = 0.542; Figure 3b) was not significantly different between cells treated with microvesicles from non‐tobacco users and e‐cigarette users. However, p‐eNOS (Ser1177) expression (28.9 ± 6.2 vs. 45.8 ± 9.0 a.u.; *F* = 10.4; *P *< 0.001; Figure 3c) was significantly lower in cells treated with microvesicles from e‐cigarette users compared with non‐tobacco users. Concordant with changes in p‐eNOS (Ser1177) expression, NO production was significantly lower (8.2 ± 0.6 vs. 9.7 ± 0.9 μmol/L; *F* = 8.3; *P* = 0.001; Figure 3d) in cells treated with microvesicles from e‐cigarette users compared with microvesicles from non‐tobacco users. Inhibition of endocytosis abrogated the effect of microvesicles from e‐cigarette users on p‐eNOS (Ser1177) expression (28.9 ± 26.5 vs. 38.1 ± 8.8 a.u.; *F* = 10.4; *P* = 0.01) and NO production (8.2 ± 0.6 vs. 9.6 ± 1.3 μmol/L; *F* = 8.3; *P* = 0.006) (Figure 3). There were no significant differences in expression of eNOS, p‐eNOS (Ser1177) and p‐eNOS (Thr495) and NO production between the untreated control cells and cells treated with microvesicles from non‐tobacco users [eNOS: 78.2 ± 26.1 vs. 71.4 ± 21.8 a.u., *P* = 0.632 (Figure 3a); p‐eNOS Ser1177: 53.7 ± 2.9 vs. 45.8 ± 9.0 a.u., *P* = 0.124 (Figure 3b); p‐eNOS (Thr495): 230.9 ± 45.6 vs. 229.2 ± 26.5 a.u., *P* = 0.932 (Figure 3c); NO: 9.5 ± 0.6 vs. 9.7 ± 0.9 μmol/L, *P* = 0.441 (Figure 3d)].

## 4 DISCUSSION

The primary new finding of this study is that circulating microvesicles from users of e‐cigarettes promote a pathological endothelial cell phenotype characterized by increased endothelial inflammation and reduced endothelial NO production. It is well established that these adverse changes in endothelial cell function contribute significantly to the initiation and development of vascular disease (Forstermann & Munzel, 2006; Libby et al., 2009). The results of the present study extend previous findings demonstrating adverse effects of endothelial cell‐derived microvesicles on human cerebral microvascular endothelial cells (Cardenas et al., 2023) and further implicate the general circulating microvesicle milieu as mediators of e‐cigarette use‐associated vascular risk.

Promotion of a pro‐inflammatory vascular endothelium is a precipitating cause of atherosclerosis. Increased endothelial cell production and release of the pro‐inflammatory cytokines IL‐6 and IL‐8 heighten vascular susceptibility to dysfunction and disease (Mudaliar et al., 2014). In the present study, microvesicles isolated from e‐cigarette users induced significantly higher endothelial cell release of IL‐6 and IL‐8, in vitro, compared with microvesicles from non‐smokers. Concordantly, cellular NF‐κB activation was markedly higher in cells treated with microvesicles from the e‐cigarette users. Nuclear factor‐κB is the primary transcription factor regulating both IL‐6 and IL‐8 production and release (Baker et al., 2011; Brasier, 2010; Xue et al., 2013). As expected, when we blocked NF‐κB activation using an IκB kinase (IKK) inhibitor, the increase in endothelial cell IL‐6 and IL‐8 production, induced by microvesicles from the e‐cigarette users, was abolished. Inhibition of IKK restricts NF‐κB translocation to the nucleus for phosphorylation and, in turn, activation (Liu et al., 2017). Thus, it appears that the pro‐inflammatory endothelial effect of microvesicles from e‐cigarette users is mediated by enhanced NF‐κB activation. Future studies are needed to elucidate the mechanisms by which microvesicles associated with e‐cigarette use upregulate NF‐κB activation. We (Hijmans et al., 2019) have previously demonstrated microvesicle‐induced changes in specific intracellular microRNAs (miRNAs), miR‐146a and miR‐181b, known to be involved in the regulation of NF‐κB activation. It is plausible that microvesicles from e‐cigarette users negatively alter the expression of these miRNAs, fostering increased activation of NF‐κB. From a clinical perspective, the microvesicle‐induced increase in endothelial cell production and release of IL‐6 and IL‐8 might underlie the chronic low‐grade systemic inflammation reported in e‐cigarette users (Singh et al., 2019).

Reduced endothelial NO production renders the vasculature highly susceptible to atherogenesis and atherosclerotic vascular events (Balakumar et al., 2012; Matthys & Bult, 1997). Furthermore, eNOS is a highly preserved and constitutively expressed endothelial enzyme. In addition to its central importance in regulating vascular tone through NO production and endothelin‐1 inhibition, eNOS activation is involved in suppressing vascular smooth muscle proliferation, inhibiting platelet aggregation and preventing leucocyte adhesion to the vessel wall (Forstermann & Munzel, 2006; Huang, 2009; Matthys & Bult, 1997). Thus, blunted eNOS activation is a major causal factor in endothelial cell dysfunction and the development of atherosclerosis and thrombosis (Forstermann & Munzel, 2006). In the present study, circulating microvesicles harvested from e‐cigarette users adversely affected eNOS activation and, in turn, NO production. Although the eNOS expression was not altered, the expression of p‐eNOS (Ser1177) was ∼65% lower in HUVECs treated with microvesicles from e‐cigarette users compared with non‐smokers. Phosphorylation is the primary post‐translational modification regulating eNOS enzyme activity (Heiss & Dirsch, 2014), and phosphorylation at the Ser1177 site confers the greatest activation of eNOS (Heiss & Dirsch, 2014). Consistent with reduced p‐eNOS (Ser1177) expression, NO production was significantly lower (∼20%) in HUVECs treated with microvesicles from the e‐cigarette users, as a consequence of diminished eNOS activation. Interestingly, p‐eNOS (Thr495) expression was not affected by the microvesicles from e‐cigarette users. Phosphorylation of Thr495 limits eNOS activity (Fleming et al., 2001; Heiss & Dirsch, 2014; Mount et al., 2007). Thus, the negative effect of e‐cigarette‐related extracellular microvesicles on eNOS activity appears to be mediated primarily through suppression of phosphorylation at the Ser1177 site. Future studies are needed to confirm this specificity and elucidate the mechanism of reduced phosphorylation.

The ability of microvesicles to be internalized and transfer their content to recipient cells is a key factor underlying their cellular effects (Mause & Weber, 2010). Microvesicles contain various proteins, nucleic acids (mRNA, miRNAs and small RNA), and lipids that, depending on the cargo signature, can dramatically affect gene expression and, in turn, induce phenotypic changes in target cells (Das & Halushka, 2015; Hulsmans & Holvoet, 2013). Although we did not determine microvesicle cargo in the present study, inhibition of endocytosis prevented the negative effects of microvesicles from e‐cigarette users on endothelial cell inflammation and NO production. Considering that extracellular vesicles can be internalized by cells via distinct endocytic pathways, we blocked clathrin‐dependent endocytosis, macropinocytosis and lipid raft mediated endocytosis non‐selectively (Mulcahy et al., 2014). Our findings provide new insight regarding how e‐cigarette‐related microvesicles affected intracellular inflammatory and NO proteins in recipient cells.

There are a few experimental considerations regarding the present study that deserve mention. Firstly, microvesicle characterization was not confirmed after ultracentrifugation in this study. Therefore, it is not possible to dismiss the possibility that the effects observed here stem solely from microvesicles; they might also originate from cellular debris and other fractions of extracellular vesicles. Future studies should be conducted to verify the characterization of microvesicles according to updated guidelines. Secondly, with any cross‐sectional human study the possibility that genetic and/or lifestyle behaviours might have influenced the results of our group comparisons cannot be dismissed definitively. We excluded adults with overt cardiometabolic disorders and disease limiting the influence of factors secondary to e‐cigarette use on our results. Thirdly, our sample size, although sufficient to detect group differences, was not sufficient to detect differences within the e‐cigarette‐using group related to the vaping brand/flavour/delivery system or possible differences between males and females. Thus, our results should be viewed in the general context of e‐cigarette use. Future studies should characterize e‐cigarette devices separately to determine whether there are any differences in extracellular microvesicle populations according to different devices (wattage and nicotine concentration). Fourthly, our in vitro studies involved the use of HUVECs, potentially raising a concern that the expression of proteins and the function of venous endothelial cells in culture might not be representative of arterial endothelial cells and, in turn, might have limited impact regarding arterial diseases, such as myocardial infarction and stroke. However, the expression of NF‐κB and eNOS has been shown to be similar in endothelial cells acquired from an artery and a vein in humans (Silver et al., 2010). Lastly, the in vitro nature of the present study precludes definitive translational statements regarding clinical risk. However, it is important to note that changes in NF‐κB‐induced inflammation and impaired eNOS activation have been linked to increased cardiovascular risk and outcome (Kooistra et al., 1994; Maccallini et al., 2017).

In conclusion, the results of this study demonstrate that circulating microvesicles from users of e‐cigarettes induce a pro‐inflammatory, NO‐impaired endothelial cell. Increased NF‐κB activation and inflammatory cytokine production, in addition to diminished eNOS activity and NO production, are hallmark aetiological factors in the development of endothelial dysfunction and, in turn, vascular disease. Circulating microvesicles might represent novel systemic mediators of vascular risk and disorders associated with e‐cigarette use.

## AUTHOR CONTRIBUTIONS

Conception and design of the work: Nicholas G. Evanoff, Vinicius P. Garcia, Michael Kotlyar and Donald R. Dengel. Data acquisition, analysis and interpretation: Nicholas G. Evanoff, Donald R. Dengel, Kelly A. Stockelman, Hannah Fandl, Noah M. DeSouza, Jared J. Greiner, Sheena R. Dufresne, Michael Kotlyar and Vinicius P. Garcia. All authors contributed to the construction of the manuscript, approved the final version of the manuscript and agree to be accountable for all aspects of the work in ensuring that questions related to the accuracy or integrity of any part of the work are appropriately investigated and resolved. All persons designated as authors qualify for authorship, and all those who qualify for authorship are listed.

## CONFLICT OF INTEREST

The authors declare no conflicts of interest.

## Supporting information


**Supplementary Figure S1**. (a) Representative histogram of particle size distribution (0.2–0.8 μm). (b) Representative Scatterplot showing microvesicle identification (blue box) within designated size range.

**Supplementary Figure S2**. The cytotoxicity of each inhibitor was assessed by MTT assay. Human umbilical vein endothelial cells (HUVECs) were treated for 3 h with (1) media (untreated cells); (2) DMSO 0.1% (vehicle); (3) DMSO 50% (positive control); (4) [5‐(*N*‐ethyl‐*N*‐isopropyl) amiloride (0.8 μM), filipin (1.5 μM) and chlorpromazine (7 μM)] (endocytosis inhibitors); and (5) IKK inhibitor VII (400 nM) (IKK‐16). Experiments were repeated four times (*n* = 4), with each treatment in triplicate. Data are presented as the mean ± SEM.

## Data Availability

Study data will be made available upon reasonable request.
