# Supplementary figures and images for: Circulating extracellular microvesicles associated with electronic cigarette use increase endothelial cell inflammation and reduce nitric oxide production
Source: Exp Physiol. 2024 Aug 2;109(9):1593–603. doi: 10.1113/EP091715 (PMC11363099; doi:10.1113/EP091715)

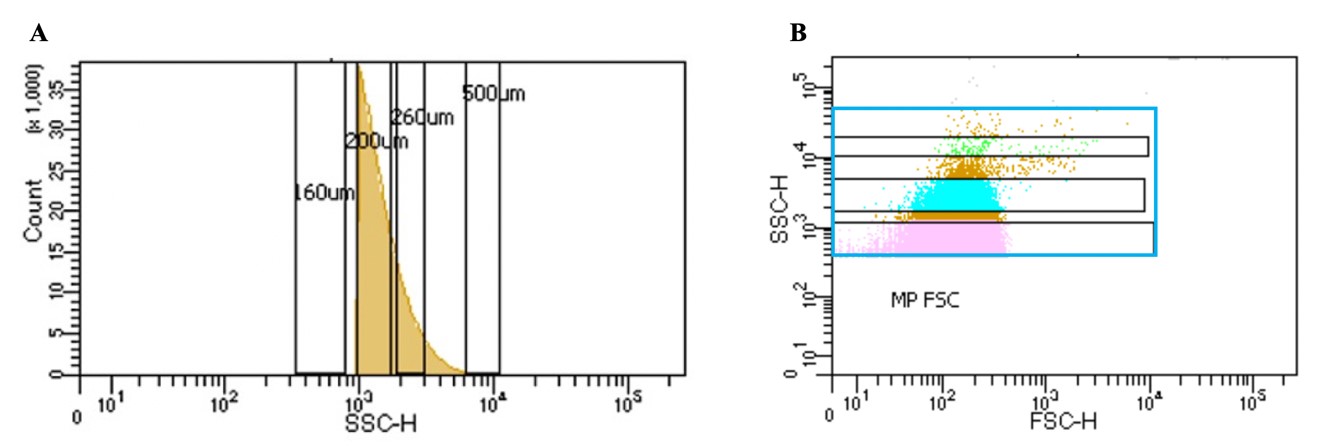

Supplement: Supplementary file 1 — Supplementary Figure S1. (a) Representative histogram of particle size distribution (0.2–0.8 μm). (b) Representative Scatterplot showing microvesicle identification (blue box) within designated size range. [file EPH-109-1593-s002.jpg]

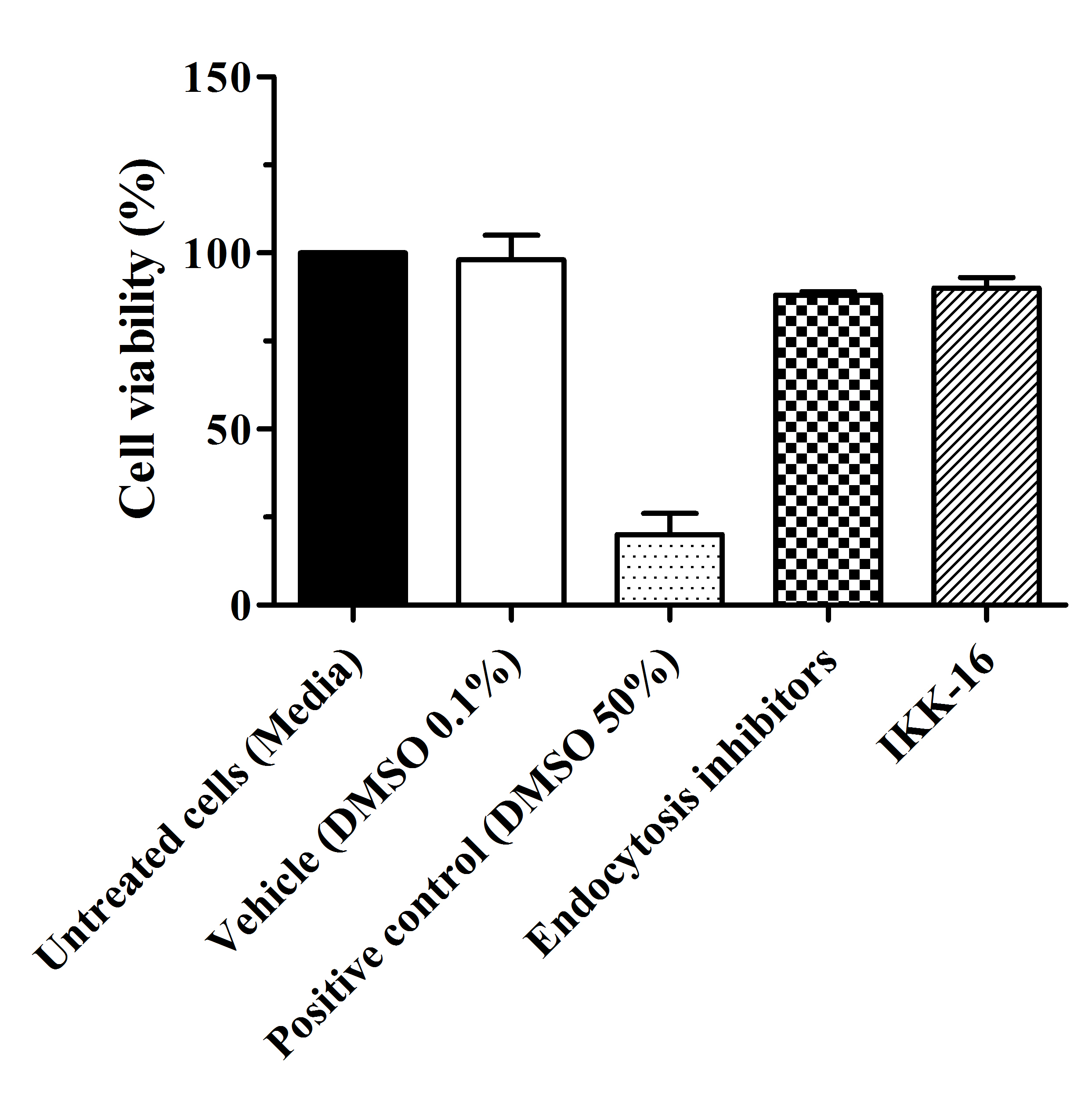

Supplement: Supplementary file 2 — Supplementary Figure S2. The cytotoxicity of each inhibitor was assessed by MTT assay. Human umbilical vein endothelial cells (HUVECs) were treated for 3 h with (1) media (untreated cells); (2) DMSO 0.1% (vehicle); (3) DMSO 50% (positive control); (4) [5‐(N‐ethyl‐N‐isopropyl) amiloride (0.8 μM), filipin (1.5 μM) and chlorpromazine (7 μM)] (endocytosis inhibitors); and (5) IKK inhibitor VII (400 nM) (IKK‐16). Experiments were repeated four times (n = 4), with each treatment in triplicate. Data are presented as the mean ± SEM. [file EPH-109-1593-s001.jpg]
